# A first meta-analysis study on body weight prediction method for beef cattle based on digital image processing

**DOI:** 10.5455/javar.2024.k760

**Published:** 2024-03-31

**Authors:** Frediansyah Firdaus, Bayu Andri Atmoko, Alek Ibrahim, Tristianto Nugroho, Endang Baliarti, Panjono Panjono

**Affiliations:** 1 Research Center for Animal Husbandry, National Research and Innovation Agency, Cibinong Science Center, Bogor, Indonesia; 2 Department of Animal Production, Faculty of Animal Science, Universitas Gadjah Mada, Yogyakarta, Indonesia

**Keywords:** Body weight, prediction, meta-analysis, beef cattle, digital image

## Abstract

**Objective:**

This study aimed to develop a method for predicting the body weight of beef cattle using meta-analysis based on digital image processing.

**Materials and Methods:**

The meta-analysis process commenced by collecting studies with the keywords “beef cattle,” “correlation,” “digital image,” and “body weight” from Google Scholar and Science Direct. The obtained studies were reviewed papers based on their titles, abstracts, and content, and then categorized by authors, year, country, sample size, and correlation coefficient. A digital image of body measurements used included wither and hip height, chest depth, heart girth, body length, and top view. The statistical analysis was conducted by calculating effect sizes using the correlation coefficient and sample sizes.

**Results:**

The results of the meta-analysis, based on 3,017 cattle from 13 selected studies, showed the highest and lowest correlation coefficients for the top view variable and hip height. Based on cattle breed, significant differences (*p* < 0.05) were observed in the wither height variable with correlation coefficients of 0.94, 0.79, and 0.66 for Hanwoo, Holstein, and Simmental, respectively. Based on sex, significant differences (*p* < 0.05) were seen in the wither height variable, with correlation coefficients of 0.73 for males and 0.90 for females, while for hip height, the values were 0.70 and 0.87, respectively.

**Conclusion:**

In conclusion, to achieve the best accuracy in predicting the body weight of beef cattle based on a digital image, the top view variable can be used. However, for ease of field experimentation, body length or chest depth can also be used while taking breed and sex categories into the model.

## Introduction

High-quality protein-rich meats from the relatively low-nutrient feed are produced from beef cattle and are not suitable for other species [1]. Therefore, strategies are required to improve the efficiency of beef cattle production, such as monitoring body weight, which is a crucial indicator in livestock production management. It includes feed formulation processes, performance analysis of male cattle, the basis for estimating livestock drug usage, growth and nutrition evaluation, health monitoring, and the determination of buying and selling prices of cattle, as well as analyzing their growth characteristics [2–3]. Farmers currently measure the body weight of cattle using digital scales. However, the limitations of this method include additional costs for scale purchases. These limitations potentially cause stress to the animals, endanger the lives of farmers, and require additional labor [4], specifically for small-scale farms and extensive livestock production systems. According to Firdaus et al. [5], another issue is that small-scale or rural farmers often lack livestock weighing facilities, leading to the assessment of body weight through subjective visual methods relying on experience. An alternative method is to predict cattle body weight using the digital image-based method of body measurement through computer vision.

According to Dohmen et al. [6], the most commonly used features for digital image-based body weight prediction are body length (11 studies), hip height (10 studies), and wither height (9 studies). The strategy uses computer vision as a non-contact alternative [7] and can be applied due to the significant correlation between body size and body weight. The body size of livestock is estimated through the analysis of biometric indices from digital images [8], followed by specific modeling to generate a prediction of body weight. However, no previous study summarizes the results of cattle body weight prediction using digital image methods through quantitative methods. Wang et al. [9] reported differences among studies, such as the relatively diverse number of livestock samples, the use of different species and breeds, and inconsistent use of measurement and outcome (O) metrics including root mean squared error, accuracy, and correlation coefficients. Other differences include experimental settings, variations in calibration methods, and factors affecting technology acceptance by producers. The use of meta-analysis is expected to address the differences in predicting cattle body weight, given the advantages of meta-analysis in summarizing, reviewing, and synthesizing studies quantitatively [10].

Based on the above, there is a need to summarize various studies on cattle body weight prediction, considering the differences in conditions. Therefore, this study aimed to develop a method for predicting cattle body weight using a digital image-based meta-analysis method categorized by cattle breed and sex.

## Materials and Methods

### Ethical Approval

This study, being a meta-analysis, does not necessitate ethical approval.

### Study design

**Figure 1.**
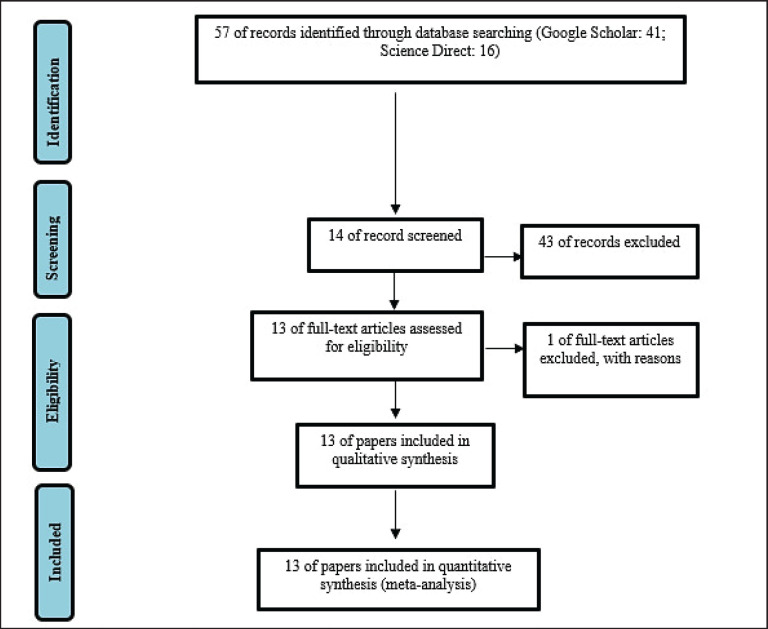
Flow chart systematic review and meta-analysis of body weight prediction based on digital image processing.

This study used a systematic review method to identify studies suitable for further meta-analysis (Figure 1). This systematic review process included gathering studies related to the prediction of beef cattle body weight using the digital image method from Google Scholar and Science Direct databases. The studies were collected using keywords based on the PICO concept, namely population: beef cattle; intervention: correlation; comparison: digital image; and O: body weight. After relevant studies were obtained, the titles and abstracts were reviewed, and information was abstracted. Data were tabulated based on authors, publication year, country of study, sample size, and correlation coefficients. The input correlation coefficients represented the relationship between body measurement and body weight based on the digital image, including wither and hip height, body length, and top view, as well as chest depth and heart girth, and top view. The definitions of each body linear measurement are as follows: 1) wither height: the perpendicular distance from the highest point of the wither behind the hump to the ground parallel to the front legs; 2) hip height: the perpendicular distance from the highest point of the hip bone to the ground parallel to the hind legs; 3) body length: the distance from the shoulder hump to the tail bone end; 4) chest depth: vertical distance from the back to the floor of the chest at the shallowest part; 5) heart girth: encircling a measuring tape around the chest behind the hump; 6) top view: capturing the back of cattle from above. The selected studies met the criteria as outlined in Table 1.

### Statistical analysis

Statistical analysis was conducted by calculating the effect size using sample size data and correlation coefficient values with the OpenMEE software [11]. A 95% confidence interval was utilized, and heterogeneity in effect size estimation was assessed using Cochran‘s Q and the *I*^2^ statistic, with an *I*^2^ value of 25%, 50%, and 75% indicating low, moderate, and high heterogeneity. Meta-analysis used a random-effects model, and the cumulative effect size was transformed into correlation coefficients, followed by data interpretation. The use of the random effect model was because the research data was quite diverse and it was necessary to consider variability between studies, so that a wider confidence interval could be obtained compared to the fixed effect model. Correlation strength was interpreted as strong when |r| ≥ 0.50. Furthermore, subgroup analysis was performed to investigate the reasons for heterogeneity in the categorization of breed and sex [12].

**Table 1.** Study inclusion/exclusion criteria.

Inclusion	Exclusion
In English	Language limitation: not in English
Any breed of beef cattle	Study neither performed on beef cattle
Reported (sample size and coefficient correlation)	No reported
Full text of publication obtained	Full text unavailable
Digital image prediction study	Wrong type of study

## Results

Through the systematic review process, 57 studies related to the theme were obtained from Google Scholar and Science Direct databases. After checking the titles and abstracts, a total of 43 studies were excluded for various reasons, including not including beef cattle, full-text unavailability, and prediction models not using the digital image method. Only 14 full studies were deemed suitable for the subsequent meta-analysis, but 1 study was excluded because it did not report correlation coefficient values (Fig. 1). Meta-analysis of digital image data yielded 5,382 samples from 3,017 cattle, as shown in Table 2. These cattle came from various stages of production, such as weaning, rearing, stockering, and feedlot phases. The majority of the study originated in Brazil and Turkey, with percentages of 30.8% each, making up a total of 61.6% in this analysis.

Digital body size images for the body weight prediction process were obtained in various ways, such as by capturing images from the top view [14], lateral view [15,16], and laser scanning [17]. Meta-analysis results of correlations between various body measurements and cattle body weight based on digital image methods showed the highest and lowest correlation coefficient estimates for the top view and hip height prediction methods (Table 3), respectively, with a difference of up to 0.12. The highest heterogeneity was observed in the hip height variable, while the lowest was in chest depth and hip height. These results indicated that using chest depth and hip height to predict cattle body weight yielded relatively consistent correlation coefficient values.

The studies for the best body measurement to predict cattle body weight showed three classification categories of correlation coefficient values. Classification c (best) was the top view; classification b included body length, wither height, chest depth, and hip height; and classification a was hip height. These results recommended the use of top-view body measurements for predicting cattle body weight using digital image-based methods. The study supports the values in Table 3, where hip height had the lowest correlation coefficient, consistent with the results of Stajnko et al. [23] that WH measurements are stronger than HH measurements.

### Meta-analysis category based on breed and sex

**Figure 2.**
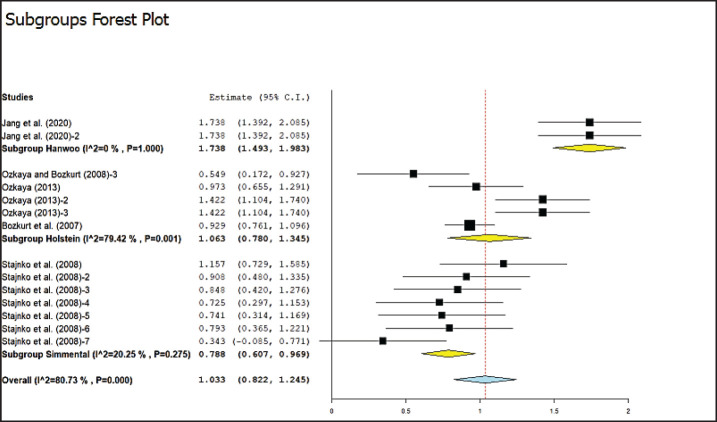
Meta-analysis of the correlation between shoulder height and body weight of cattle based on digital image with cattle breed category.

The results of the correlation analysis between body measurements and cattle body weight for categorization based on breed and sex are shown in Tables 4 and 5, respectively. This result showed that categorization based on cattle breed yielded significantly different results, particularly for the correlation between shoulder height and body weight, as indicated in Table 4 and Figure 2. Meta-analysis results showed that the highest correlation value was observed in Hanwoo cattle with the wither height variable, which was 0.94, while the lowest correlation was in Simmental cattle with 0.66. However, no significant differences were observed among breeds for body length, chest depth, or hip height. The highest heterogeneity was found in Holstein cattle at 87.69%, suggesting that categorizing cattle by breed is necessary to obtain higher correlation coefficients, thereby affecting the prediction of body weight. The analysis of body length, chest depth, and hip height showed no significant differences in correlation with cattle body weight when categorized by breed. This indicates that categorization may not be necessary for the prediction modeling of cattle body weight when using variables, such as body length, chest depth, and hip height. However, categorization is required to obtain better prediction results when the wither height variable is used.

**Table 2.** Database of studies of predicting body weight of beef cattle used in meta-analysis based on digital image processing.

No	Authors	Year	Country	Breed	Sex	*N* (head)
1	Jang et al. [7]	2020	South Korea	Hanwoo	F	35
2	Gomes et al. [8]	2016	Brazil	Black Angus, Nellore	M	35
3	Ozkaya and Bozkurt [13]	2008	Turkiye	Holstein, Brown Swiss, Crossbred	M	140
4	Cominotte et al. [14]	2020	Brazil	Nellore	M	62
5	Ozkaya et al. [15]	2015	Poland	Limousin	M	56
6	Bozkurt et al. [16]	2017	Turkiye	Brown Swiss, Holstein	M	40
7	Sousa et al. [17]	2018	Brazil	Nellore	M	107
8	Weber et al. [18]	2020	Brazil	Nellore	M	19
9	Ozkaya [19]	2013	Turkiye	Holstein	F	41
10	Bozkurt et al. [20]	2007	Turkiye	Holstein	M	140
11	Nilchuen et al. [21]	2021	Thailand	Crossbred (Brahman, Charolais)	F	160
12	Miller et al. [22]	2019	Scotland	Angus, Limousin, Simmental, Charolais	M	2.158
13	Stajnko et al. [23]	2008	Slovenia	Simmental	M	24

BM= body measurement; M= male; F= female.

**Table 3.** Meta-analysis of the correlation between various body measurements and beef cattle body weight based on digital image processing.

Variable	Coefficient correlation			Heterogeneity	*N* (head)
	Estimate	Lower	Upper	*I* ^2^	
BL	0.82^b^	0.75	0.88	82.42%	604
WH	0.79^b^	0.71	0.85	81.31%	737
CD	0.86^b^	0.81	0.90	69.39%	484
TV	0.89^c^	0.86	0.91	83.41%	3.276
HH	0.77^a^	0.68	0.84	69.85%	431
HG	0.88^b^	0.41	0.98	98.47%	300

*N* = total sample; ^a,b,c^ different letters in the diagram indicate significant differences (*p* < 0.05) based on the meta-analysis subgroups; BL= body length; WH = wither height; CD = chest depth; TV = top view; HH = hip height; HG = heart girth.

**Figure 3.**
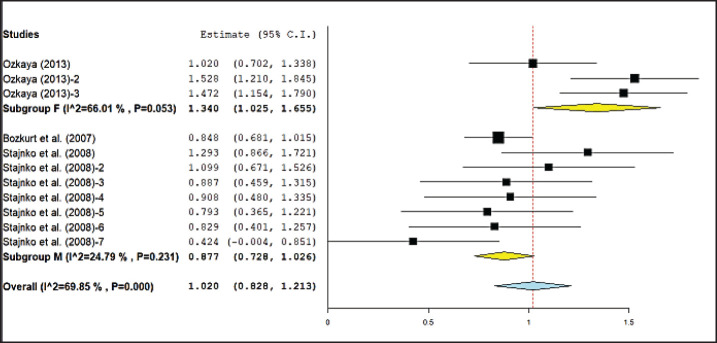
Meta-analysis of the correlation between hip height and body weight of cattle based on digital image with sex category.

**Figure 4.**
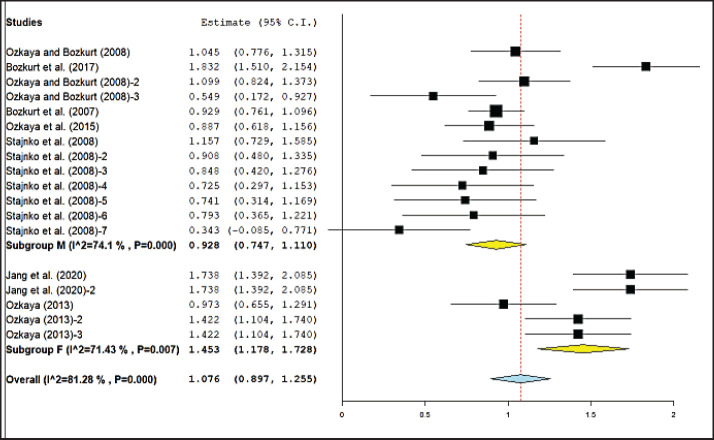
Meta-analysis of the correlation between shoulder height and body weight of cattle based on digital image with sex category.

A comprehensive summary of meta-analysis results regarding the relationship between digital image-based body measurement and body weight in different cattle sexes is shown in Table 5. The result showed that body measurements had a significant effect (*p* < 0.05) on both male and female cattle body weight. However, no significant differences were recorded between subgroups of male and female cattle in wither height and hip height measurements. Figures 3 and 4 illustrate forest plots showing meta-analysis results found for the effect of hip height and shoulder height on cattle body weight based on digital image categorization by sex. The highest estimated correlation coefficient was identified in the method based on the wither height of female cattle (0.90), while the lowest was for the hip height of bulls (0.70). High heterogeneity (*I*^2^ > 50%) was observed in all body sizes of male and female cattle, except for body length in females (*I*^2^ = 48.16%, moderate heterogeneity) and hip height in male cattle (*I*^2^ = 24.79%, low heterogeneity).

**Table 4.** Meta-analysis of the correlation of various body measurements based on digital image on body weight with cattle breed category.

Variable	Coefficient correlation			Heterogeneity	*N* (head)
	Estimate	Lower	Upper	*I*^2^ (%)	
Body length					
Hanwoo	0.83	0.67	0.92	56.62%	70
Holstein	0.80	0.70	0.88	74.01	293
Wither height					
Hanwoo	0.94^c^	0.90	0.96	0%	70
Holstein	0.79^b^	0.65	0.87	79.42%	293
Simmental	0.66^a^	0.54	0.75	20.25%	168
Chest depth					
Hanwoo	0.91	0.85	0.94	0%	70
Holstein	0.83	0.58	0.94	87.69%	123
Hip height					
Holstein	0.83	0.69	0.92	85.68%	263
Simmental	0.71	0.60	0.80	34.64%	168

*N* = total sample; ^a,b,c^ different letters in the diagram indicate significant differences (*p* < 0.05) based on the meta-analysis subgroups.

**Table 5.** Meta-analysis of the correlation of various digital image-based body measurements with body weight by sex category.

Variable	Coefficient correlation			Heterogeneity	*N* (sample)
	Estimate	Lower	Upper	*I*^2^ (%)	
Body length					
Male	0.81	0.67	0.89	88.34%	411
Female	0.85	0.78	0.89	48.16%	193
Wither height					
Male	0.73^a^	0.63	0.80	74.10%	544
Female	0.90^b^	0.83	0.94	71.43%	193
Chest depth					
Male	0.87	0.77	0.92	59.34%	131
Female	0.86	0.78	0.91	75.73%	353
Hip height					
Male	0.70^a^	0.62	0.77	24.79%	308
Female	0.87^b^	0.77	0.93	66.01%	123

*N* = total sample; ^a,b^ different letters in the diagram indicate significant differences (*p* < 0.05) based on the meta-analysis subgroups.

## Discussion

The development of cattle body weight prediction based on a non-contact digital image measurement is beneficial for improving animal well-being and livestock production management, as well as saving monitoring time. The process of cattle body weight prediction based on digital images found in this study has several steps, which include the collection of digital image-based cattle body measurements using computer vision methods [24]. The obtained images are processed to be used as predictor variables in the prediction of body weight in statistical models. Finally, modeling was conducted to generate equations for predicting cattle body weight, which were then integrated into a system for showing weight prediction results.

The previous studies summarized in this meta-analysis showed that digital image-based body measurements can be used to predict cattle body weight, with a correlation coefficient ranging from 0.77 to 0.89. However, categorization based on breed has the potential to increase correlation coefficient values to as high as 0.96, while sex can potentially increase the value to 0.94. The best body measurements for predicting body weight are top view, chest girth, and wither height. The use of a top-view image can be challenging for application in traditional and small-scale farming, but heart girth and wither height may be more suitable for such consumers. Furthermore, the use of top-view digital images, captured from above using specific cameras, has great potential in large-scale farming, where there is typically more investment capacity for acquiring such equipment. For small-scale farmers, body length, height, or chest depth are quite effective, with the potential for correlation coefficient values to reach 0.96 and support categorization based on breed and sex. These body measurements were obtained with the assistance of deep learning algorithms. Weight estimation is then carried out by computer vision methods, with linear regression algorithms being the most commonly used modeling method.

Categorization based on breed and sex can enhance the reliability of prediction, with correlation coefficients reaching up to 0.96. The breed is considered an appropriate categorization indicator in predicting cattle body weight due to the distinct phenotypic and conformational characteristics of each cattle breed [25]. Future studies should consider the use of machine learning-based algorithms, as reported by Ruchay et al. [26]. This is because the extra tree regressor algorithm, using morphometric measurements and cattle age, provided better results than regression for predicting body weight. Other methods, such as predictive methods like ANN, have shown improved body weight prediction results [27]. Focus should also be placed on addressing the limitations of depth cameras, as reported by Xiong et al. [28], which require 1–5 min to obtain high-quality images. The strategy modifies and improvises the image capture process through video recording, which is believed to enable faster data collection and accommodate the natural movement speed of livestock. In addition, the cameras used should be resistant to dust, moisture, and potential damage caused by livestock. Technology practicality is also needed, including various cattle breeds, ages, diverse body condition scores, production phases, and color patterns in different environments.

In future research development, researchers can focus on developing predictions of cattle weight based on two levels of target consumers. The first is for large-scale farms and medium- and small-scale farms. For large-scale farms, automatic segmentation of digital images can use a top view or a combination of three types of body measurements, namely shoulders, chest, and hips, through a 3D approach. However, Hou et al. [29] reported that there were several problems in developing a digital image-based cattle body weight prediction method. For example, when a cattle changes its body posture, such as raising or lowering its head, the extreme points of the back line do not always coincide with the position of the withers. This causes some calculation errors. To obtain better accuracy values, it is also necessary to consider environmental factors, feed consumption, and weight during the growth period [30]. Meanwhile, on small-scale farms, you can use a 2D approach, using heart girth, body length, wither height, and chest depth.

## Conclusion

In conclusion, the top view variable could be used to achieve the highest accuracy in predicting the body weight of beef cattle based on digital image processing. However, for field experiments that required portability, body length, and chest depth methods are more suitable with categorization based on breed and sex.

## List of Abbreviations

RMSE, Root mean squared error; O, outcome.
